# Filtering genetic variants and placing informative *priors* based on putative biological function

**DOI:** 10.1186/s12863-015-0313-x

**Published:** 2016-02-03

**Authors:** Stefanie Friedrichs, Dörthe Malzahn, Elizabeth W. Pugh, Marcio Almeida, Xiao Qing Liu, Julia N. Bailey

**Affiliations:** Department of Genetic Epidemiology, University Medical Center, Georg-August University Göttingen, Göttingen, Germany; Center for Inherited Disease Research, Institute of Genetic Medicine, Johns Hopkins School of Medicine, Baltimore, MD USA; South Texas Diabetes and Obesity Institute, University of Texas Rio Grande Valley, Brownsville, TX USA; Department of Obstetrics, Gynecology, and Reproductive Sciences, Department of Biochemistry and Medical Genetics, Faculty of Health Sciences, University of Manitoba, Winnipeg, MB Canada; Children’s Hospital Research Institute of Manitoba, Winnipeg, MB Canada; Department of Epidemiology, Fielding School of Public Health, University of California, Los Angeles, Los Angeles, CA USA; Epilepsy Genetics/Genomics Laboratory, West Los Angeles Veterans Administration, Los Angeles, CA USA

## Abstract

High-density genetic marker data, especially sequence data, imply an immense multiple testing burden. This can be ameliorated by filtering genetic variants, exploiting or accounting for correlations between variants, jointly testing variants, and by incorporating informative *priors. Priors* can be based on biological knowledge or predicted variant function, or even be used to integrate gene expression or other omics data. Based on Genetic Analysis Workshop (GAW) 19 data, this article discusses diversity and usefulness of functional variant scores provided, for example, by PolyPhen2, SIFT, or RegulomeDB annotations. Incorporating functional scores into variant filters or weights and adjusting the significance level for correlations between variants yielded significant associations with blood pressure traits in a large family study of Mexican Americans (GAW19 data set). Marker rs218966 in gene *PHF14* and rs9836027 in *MAP4* significantly associated with hypertension; additionally, rare variants in *SNUPN* significantly associated with systolic blood pressure. Variant weights strongly influenced the power of kernel methods and burden tests. Apart from variant weights in test statistics, *prior* weights may also be used when combining test statistics or to informatively weight *p* values while controlling false discovery rate (FDR). Indeed, power improved when gene expression data for FDR-controlled informative weighting of association test *p* values of genes was used. Finally, approaches exploiting variant correlations included identity-by-descent mapping and the optimal strategy for joint testing rare and common variants, which was observed to depend on linkage disequilibrium structure.

## Background

With the availability of very dense genetic marker data sets, such as sequence data, even large association studies can become underpowered. This raises the need to filter, or prioritize, or jointly test genetic variants.

Filters or *priors* on genes may be derived from methylation or expression data if available in the same individuals. Alternatively, one may use external information. Recently, multiple annotation tools have become available using several databases and algorithms that predict functional effects of genetic variants. Commonly used are, for example, ANNOVAR (Annotate Variation) [1], VariantTools [2], PolyPhen [3], SIFT (Sorting Intolerant From Tolerant) [4], ENCODE (Encyclopedia of DNA Elements) [5], RegulomeDB [6], CADD (Combined Annotation-Dependent Depletion) [7], or Gerp++ [8]. Tools like ANNOVAR additionally provide variant annotation to genes and to regions such as conserved regions among species, predicted transcription factor binding sites, and segmental duplication regions. Many of the above-listed tools also provide information on regulatory elements that control gene activity. This article demonstrates that functional scores can contribute to the success of association studies. Simultaneously, functional scores may differ substantially between databases and prediction tools as they can be based on different functional aspects. Additionally, variant annotations to chromosomal positions continue to be updated with the National Center for Biotechnology Information (NCBI) [9] human genome build as standard. Furthermore, variants can be annotated to genes based on different sources, such as ENSEMBL [10], Vega [11], GENCODE [12], and many more. Researchers also use a variety of definitions of flanking regions. Finally, genes may be grouped by function or biological pathway, again with substantial variability between data bases such as KEGG [13], Biocarta [14], or Pathway Interaction Database [15]. This article discusses approaches that filtered or prioritized genetic variants, regions, or genes. Pathway-based approaches, although also incorporating filters or *priors*, are discussed separately by Kent [16].

Many researchers filter genetic variants. The simplest forms of filters are minor allele frequency (MAF), candidate genes or variants, or considering the exome. Filters and statistical models are chosen to increase the power under a hypothetical disease model. The advent of sequencing renewed interest in disease mechanisms less frequent but more penetrant than common single nucleotide polymorphisms (SNPs) of genome-wide association studies (GWAS). This led, for example, to screening for recessive variants by examining runs of homozygosity [17, 18]. When multiple rare causal variants cluster within a gene, identity-by-descent (IBD) mapping may be more powerful than single-locus association testing [19]. IBD mapping can be used in 2-step approaches. For example, Balliu et al [20] identified regions where hypertension cases shared more segments of IBD than controls in one part of the sample. They modeled aggregate effects of each of these regions on blood pressure (BP) in the sample remainder. Aggregation tests are used especially for testing rare single-nucleotide variants (SNVs). Aggregation tests are burden tests, variance-component tests, or a combination of both, such as SKAT-O (optimal unified sequence kernel association test) (see, eg, Lee et al [21] for a review). Kernel-based approaches (see Schaid [22] for a review) such as the sequence kernel association test (SKAT) [23] are variance-component tests. Examples of genetic burden tests are T5, combined multivariate collapsing (CMC) [24], or C-α [25]; see also Santorico et al [26]. Aggregation tests can prioritize SNVs by weighting minor allele dosages in the test statistic. Typical weights account for MAF, but may also incorporate putative functional relevance of SNVs [27, 28]. Moreover, weights may be used to combine aggregation test statistics [21, 29, 30], and one may weight *p* values while controlling the false discovery rate (FDR) [31, 32]. For example, GWAS *p* values may be weighted based on functional annotations. For aggregation tests on genes, *p* value weights can be utilized to integrate gene expression or other omics data [33].

This article summarizes contributions of the Genetic Analysis Workshop (GAW) 19 group on filtering variants and placing informative *priors* (Tables 1 and 2). These investigations found that improving SNV grouping or selection can noticeably increase power. Moreover, including functional scores or gene expression data as filters or weights on variants, genes, or when combining test statistics assisted in detecting associations. Some contributions also exploited SNV correlations to increase power or improved the multiple-testing adjusted significance threshold by accounting for SNV correlations.

**Table 1:** Statistical tests and analyzed data

	Marker data	Data set	Statistical tests	Covariates	Trait(s)
*Almeida et al* [36]					
	Sequence	Family study	Single-variant regression in SOLAR	Smoking, BP medication, PC1-3, sex, age, age^2^, sex*age, sex*age^2^	Real SBP and DBP at first time point, own simulated trait for H_0_
*Liu et al* [37]					
	Chr3: GWASmp and sequence	Unrelated individuals (from family study)	Regress pairwise DBP residual difference and sum on IBD sharing status; sequence data analyses by SKAT-O	Sex, age, smoking, PC 1-3	Real DBP at first time point
*Kim and Wei* [27]					
	Sequence	Family study	Informative SNV weights in burden test T5 and SKAT; with R: seqMeta	Age, sex, smoking, BP medication	Real SBP at earliest available measurement
*Zhang et al* [28]					
	Exome sequence	Unrelated individuals (large Hispanic sample)	LRT, C-$\alpha$, CMC on informatively weighted SNV burden	None	Simulated HT status; real SBP, DBP with cutoffs for case-control status
*Malzahn et al* [30]					
	Sequence and GWASmp	Family study	SKAT with R (coxme, kinship2, QuadCompForm); strategies for joint testing of rare and common SNVs	Sex, age, sex*age; subjects not on BP medication	Real and simulated SBP at first time point
*Ho et al* [33]					
	Sequence and GWASmp	Family study, including gene expression data	Seq-aSum-VS burden test; regression on gene expression data; gene set enrichment analysis	PC1-3	Average real SBP and DBP

*BP* blood pressure, *Chr* Chromosome, *CMC* Combined multivariate collapsing, *DBP* diastolic blood pressure, *GWASmp* genome-wide association study marker panel, *HT* hypertension, *IBD* identity-by-descent, *LRT* likelihood ratio test, *PC* principal component, *SBP* systolic blood pressure, *SKAT* sequence kernel association test, *SNV* single nucleotide variant, *Seq-aSum-VS* sequential sum

**Table 2:** Filters, *priors*, and findings

	Filter	*Prior*	Conclusions	Annotation
*Almeida et al* [36]				
	Functional annotation, LD-corrected effective number of tests	None	LD-correction in WGS reduces multiple-testing burden by 85 %, significant associations: *PFH14* with SBP, *MAP4* with DBP	Location: ANNOVAR; functional annotation: PolyPhen, SIFT
*Liu et al* [37]				
	IBD sharing	None	No significances, *ZPLD1* had strongest evidence	IBD mapping: BEAGLE; functional annotation: CADD
*Kim and Wei* [27]				
	Sliding window on MAF ≤5 % SNVs	*SNV-weights:* based on MAF or regulatory importance	Significant association: *SNUPN*	Functional annotation: ENCODE, RegulomeDB, PolyPhen2
*Zhang et al* [28]				
	Genes, exome-sequence	*SNV-weights:* up-weight protein binding sites, apply direction weights	Top-ranked genes differ between weighted burden tests LRT, C-α, CMC; but good overlap with literature	ANNOVAR, variant tools; random forest classifiers assign SNVs to protein binding sites; DSSP, PSAIA, DOMINO
*Malzahn et al* [30]				
	Gene covering LD-blocks	*SNV-weights:* using MAF	SKAT: power depends on SNV weights, exploiting LD is very beneficial, optimal strategy for joint testing rare and common SNVs depends on LD structure	Haploview with HapMap data for LD-calculation
		*Overall weight:* on rare SNV variance component in SKAT		
*Ho et al* [33]				
	Rare SNVs in genes with >1 and <50 rare SNVs (MAF < 0.01)	*p value weights:* improve gene ranking	Power of burden tests improved by incorporating phenotype associated gene expression into *p* value weights	Genes: hg19; GO biological process categories

*CADD* combined annotation dependent depletion, *DBP* diastolic blood pressure, *DOMINO* database of domain–peptide interactions, *DSSP* define secondary structure of proteins, *ENCODE* encyclopedia of DNA elements, *GO* gene ontology, *IBD* identity-by-descent, *LD* linkage disequilibrium, *MAF* minor allele frequency, *PSAIA* protein structure and interaction analyzer, *SBP* systolic blood pressure, *SIFT* sorting intolerant from tolerant, *SKAT* sequence kernel association test, *SNV* single nucleotide variant, *WGS* whole genome sequence

### Materials

Analyzed data were provided by GAW 19 and included a family sample (n = 959) with extended pedigrees of Mexican Americans from the San Antonio Family Heart Study (SAFHS) and the San Antonio Family Diabetes/Gallbladder Study (SAFDS/ SAFGS) [34]. The family sample also contained 103 unrelated sequenced subjects; 259 subjects had gene expression data. This study was designed to identify low-frequency or rare variants influencing susceptibility to type 2 diabetes (T2D) as part of the T2D Genetic Exploration by Next-generation sequencing in Ethnic Samples (T2D-GENES) Consortium. Phenotypes included real and simulated longitudinal systolic (SBP) and diastolic blood pressure (DBP) and hypertension (HT) status. Available were sequence for 464 pedigree members and GWAS SNPs for all 959 subjects. Additionally, all subjects were imputed to sequence based on original genotypes and familial relationships [34]. Approaches described herein mostly analyzed imputed dosages to avoid missing genotypes and to maximize sample size. Zhang et al [28] analyzed the GAW19 sample of 1943 independent Hispanic subjects with whole exome sequence. This sample had been ascertained by T2D status. However, GAW19 provided real and simulated cross-sectional BP traits instead [35], using the same trait-simulation model as for the family study.

All approaches described herein are nonlongitudinal analyses of BP traits (SBP, DBP, or HT) in relation to minor allele dosages of sequence SNVs or genome-wide SNPs.

## Methods

Statistical methods employed by this group (see Table 1) to incorporate filters or informative *priors* are mostly based on regression models [27, 30, 33, 36, 37]; one is also based on counting methods [28]. Analyses of family data adjusted for familial dependence based on the kinship matrix. They included the familial covariance in a linear mixed model [27, 30, 36] or transformed the trait to a conditionally independent surrogate variable [33]. Analyses of independent subjects accounted for population structure (cryptic relatedness and admixture) [37] by using the programs Eigensoft [38] and Admixture [39].

### Annotating genetic variants for location and function

A variety of freely available genetic databases and highly developed software tools support annotation of location and biological function of SNVs. In our group, SNV locations were obtained by ANNOVAR [28, 36] or determined based on reference data, for example, from the Genome Reference Consortium [40] or the International Haplotype Map (HapMap) Consortium [41] [30, 37]. Reference data were also used to determine linkage disequilibrium (LD) blocks [30] with Haploview [42].

Kim and Wei [27] and Almeida et al [36] used functional annotations from ENCODE, PolyPhen or PolyPhen2, and SIFT, while Liu et al [37] used CADD. In contrast, Zhang et al [28] annotated putative protein binding sites based on 2 different algorithms using random forest classifiers [43].

### Filtering genetic variants

Not all areas of the genome were studied. Some researchers filtered the data prior to analyses. Zhang et al [28] investigated exome sequence and Almeida et al [36] molecularly functional nonsynonymous SNVs predicted by PolyPhen and SIFT. Liu et al [37] examined IBD sharing regions on chromosome 3. Malzahn et al [30] considered gene-containing LD blocks for selected candidate genes. Ho et al [33] analyzed rare SNV burden in genes containing less than 50 and more than 1 rare SNV (MAF <0.01).

### Accounting for correlations between genetic variants

An important difference between methods is that variant correlations can either be a nuisance or may be used to increase power. For example, IBD mapping exploits variant correlations. IBD mapping can be more powerful than single-locus association testing when multiple causal rare variants cluster within a gene [19]. Therefore, Liu et al [37] tested the relationship between IBD sharing status and trait differences and sums for pairs of individuals. Moreover, the power of kernel methods such as SKAT may be increased through the exploitation of variant correlations [44]. This ability can be utilized fully by analyzing LD blocks [30]. On the other hand, single-locus methods need to account for variant correlations to appropriately correct the significance level for multiple testing. Hence, Almeida et al [36] determined the effective number of independent tests by extreme value theory based on replicates of a simulated unassociated trait.

### Correcting the significance level for the number of independent tests

The significance level used with multiple testing is always an issue as too conservative a correction will cause false negatives and not correcting enough will cause false positives.

Almeida et al [36] adjusted the significance level for single locus analyses by estimating the number of independent tests [45]. A total of 1000 replicates of a quantitative phenotype with no genetic effects were simulated and tested on whole genome sequence data, using linear mixed models in SOLAR (Sequential Oligogenic Linkage Analysis Routines) [46]. The smallest *p* value per simulation run was extracted. The density of these 1000 extremely small *p* values was fitted to a theoretical beta distribution *beta*(1,*n*_*e*_) where *n*_*e*_ is the effective number of independent tests [47]; yielding the adjusted significance level ${a}^{\ast }=\frac{0.05}{n_e}$. This procedure was applied to both whole genome sequence and functional nonsynonymous SNVs.

### Identity-by-descent mapping

IBD mapping aims to detect loci sharing ancestral segments in unrelated individuals. In particular, unrelated subject-pairs with smaller trait differences are expected to share significantly more rare causative variants than pairs with larger trait differences. Liu et al [37] estimated IBD sharing segments with BEAGLE [48]. The squared trait difference (D) and squared trait sum (S) for trait DBP between pairs of unrelated subjects was regressed on IBD sharing status. This yielded parameter estimates for slopes $\left({\widehat{\beta}}_S,{\widehat{\beta}}_D\right)$ and variances (*σ*_*S*_^2^, *σ*_*D*_^2^), which were combined into an overall slope estimate $\widehat{\beta}=\left(\frac{\sigma_D^2}{\sigma_S^2+{\sigma}_D^2}\right)\ {\widehat{\beta}}_s + \left(\frac{\sigma_S^2}{\sigma_S^2+{\sigma}_D^2}\right)\ {\widehat{\beta}}_D$. Linkage was tested with test statistic $t=\frac{\widehat{\beta}}{SE\left(\widehat{\beta}\right)}$ under the null hypothesis of an overall slope of zero [37]. The significance threshold for nonindependent pairs was estimated by permutation procedure.

### *Priors* on genes and variants

Genetic *priors* can be incorporated by variant weights in aggregation tests such as burden tests or SKAT [21]. Burden tests collapse minor allele dosages *x*_*ik*_ of a set of *i* = 1, …, *m* variants into a burden score *s*_*k*_ = ∑_*i* = 1_^m^*ω*_*i*_*x*_*ik*_ per individual *k* using a priori specified variant weights *ω*_*i*_. One tests trait association with genetic burden *s*_*k*_. Although burden tests are powerful when causal SNVs have the same effect direction, SKAT is more powerful when effect directions differ or if many noncausal SNVs are included in testing [21, 49]. SKAT is based on an underlying Bayesian model that estimates a random effect per SNV [50]. Specified is a kernel matrix of genetic between-subject similarity and this kernel constitutes a *prior* on genetic model space [51]. SNV weights are incorporated in the kernel (see, eg, Malzahn et al [30]).

Typically, rarer SNVs get assigned more weight to counterbalance their reduced power compared to more frequent SNVs. Used are, for example, weights ${\omega}_j=\frac{1}{\mathrm{MA}{\mathrm{F}}_{\mathrm{j}}\left(1-\mathrm{M}\mathrm{A}{\mathrm{F}}_{\mathrm{j}}\right)}$ [52], inverse MAF weights ${\upomega}_{\mathrm{j}}=\frac{1}{\mathrm{MA}{\mathrm{F}}_{\mathrm{i}}}$, or *beta*-weights such as ω_j_ = *b*(MAF_i_) [23], where *b* is the probability density function of a *beta*(1, 25) random variable. Malzahn et al [30] compared the power of SKAT when using different SNV weights and different kernel functions that either allow or do not allow for SNV interactions in the genetic model. Alternatively, SNV weights may be based on regulatory importance [27] or protein binding effects [28].

### Incorporating functional information into variant weights

Kim and Wei [27] categorized SNVs according to RegulomeDB and PolyPhen2 functional relevance scores. SNV weights were defined based on *f*(*s*) = *S*^2^ where *s* equaled the reverse order of categories, namely s = 6, 5, 4, 3, 2, 1 for category 1 (“most likely affecting binding and expression”) to category 6 (“not functionally relevant”). Kim and Wei [27] tested rare SNVs jointly, in sets defined by sliding windows of 4 kb size, for association with SBP. They compared the power of SNV weighting schemes in SKAT (${\omega}_j=\sqrt{f\left({s}_j\right)}$ versus *ω*_*j*_ = *b*(MAF_j_)), and burden test T5 (*ω*_*j*_ = *f*(*s*_*j*_) versus ${\omega}_j=\frac{1}{\mathrm{MA}{\mathrm{F}}_{\mathrm{j}}\left(1-\mathrm{M}\mathrm{A}{\mathrm{F}}_{\mathrm{j}}\right)}$). SKAT and T5 provide analytical asymptotically exact *p* values with good small sample size behavior.

Zhang et al [28] used a likelihood ratio test (LRT) [53] to test if the proportion of subjects with an informatively weighted minor allele burden exceeding a given threshold differed between HT cases and controls. *P* values were obtained by permutation procedure. SNV weights *ω*_*i*_ accounted for putative effect direction and distinguished between functional SNVs in binding-sites (|*ω*_*i*_| = 10), not in binding-sites (|*ω*_*i*_| = 5), and nonfunctional SNVs (|*ω*_*i*_| = 1). The informatively weighted LRT was compared with C-α and CMC burden tests.

### Optimal joint testing of rare and common variants

When not filtering for rare or common SNVs, optimal joint testing of both becomes an issue. Suppose, one computed 2 SKAT statistics, Q_rare_ and Q_common_, separately on rare SNVs and common SNVs, in the same region of interest, for the same trait, based on the same genetic null model. As SKAT is a variance-component test, combining Q_rare_ and Q_common_ [29]

1 $$ \mathrm{Q}\mathrm{w}\mathrm{s}=\left(1-\lambda \right)\cdot {\mathrm{Q}}_{\mathrm{rare}}+\lambda \cdot {\mathrm{Q}}_{\mathrm{common}} $$

weights the rare SNV variance-component by overall a priori weight (1-*λ*) relative to the common SNV variance-component (see Ionita-Laza et al [29] and Malzahn et al [30] for choices of *λ*). The weighted sum test (1) is another way of structuring a *prior* in SKAT. Note that Q_rare_ and Q_common_ may use different kernel functions or different SNV weights. Malzahn et al [30] compared this form of joint testing of rare and common SNVs with the default choice of entering *all* SNVs with appropriate weights into a *single* kernel. Exact *p* values for SKAT and weighted sum test (1) were obtained by Davies method [54]. Another investigated alternative was Fisher pooling of the correlated *p* values resulting from the separate rare SNV and common SNV SKAT statistics. Fisher pooling accounted for correlations by Satterthwaite approximation and Brown’s method ([55]; see also [29, 30]).

Note that analogously to equation (1), SKAT-O combines SKAT and burden tests with statistic *Q* = (1 − *ρ*)*Q*_*SKAT*_ + *ρQ*_*burden*_ where 0 ≤ *ρ* ≤ 1 [56].

### Informed *p* value weighting for genes

Ho et al [33] obtained gene-wise *p* values, ${p}_{\mathit{\mathsf{g}}}$_,_ for association of average BP *T* with rare SNV burden ${s}_{\mathit{\mathsf{g}}}$ in genes $\mathit{\mathsf{g}}$ that had more than 1 and less than 50 rare SNVs (MAF <0.01)

2 $$ T\sim {b}_{s,\mathit{\mathsf{g}}}\cdot {s}_{\mathit{\mathsf{g}}} $$

Restricting the number of rare SNVs avoids collapsing too many null variants. Ho et al [33] used the sequential sum test [57], which data-adaptively assigned SNV weights *ω*_*i*_ = 0, 1, − 1. Earlier, Genovese et al [31] and Roeder and Wasserman [32] had proven that informative weighting of *p* values $\frac{p_{\mathit{\mathsf{g}}}}{\nu_{\mathit{\mathsf{g}}}}$ with weights ${v}_{\mathit{\mathsf{g}}}>0,\;{\overline{v}}_{\mathit{\mathsf{g}}}=1$ maintains proper FDR control; where $\frac{p_{\mathit{\mathsf{g}}}}{\nu_{\mathit{\mathsf{g}}}}\le {\alpha}_{FDR}$ means significance. Ho et al [33] determined such weights ${v}_{\mathit{\mathsf{g}}}$ as follows. They tested if rare minor allele burden ${s}_{\mathit{\mathsf{g}}}^{*}$ (with SNV weights *ω*_*i*_ = 1, for simplicity) also associated with gene expression ${E}_{\mathit{\mathsf{g}}}$

3 $$ {E}_{\mathit{\mathsf{g}}}\Big|T \sim {b}_{E,\mathit{\mathsf{g}}}\cdot {s}_{\mathit{\mathsf{g}}}^{*}+c\cdot T $$

and further if gene expression ${E}_{\mathit{\mathsf{g}}}$ associated with trait value *T*

4 $$ T\Big|{s}_{\mathit{\mathsf{g}}}^{*} \sim {b}_{T,\mathit{\mathsf{g}}}\cdot {E}_{\mathit{\mathsf{g}}}+d\cdot {s}_{\mathit{\mathsf{g}}}^{*} $$

**Fig. 1:**
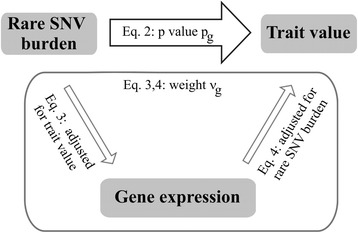
Informed *p* value weighting for genes based on conditionally independent associations between rare variant burden, gene expression, and trait. The *p* value weight ${v}_{\mathit{\mathsf{g}}}$ was defined as the product of the association strengths of rare SNV burden with gene expression and gene expression with trait value

Association tests (2) to (4) were made conditionally independent by adjusting test (3) for trait value *T* and test (4) for rare minor allele burden ${s}_{\mathit{\mathsf{g}}}^{*}$ (Fig. 1). *P* value weights ${\nu}_{\mathit{\mathsf{g}}}=\frac{\nu_{\mathit{\mathsf{g}}}^{*}}{\overline{v_{\mathit{\mathsf{g}}}^{\ast }}}$ were derived as ${\nu}_{\mathit{\mathsf{g}}}^{*}= max\left({\left(\frac{\widehat{b_{E,\mathit{\mathsf{g}}}}}{SE\left(\widehat{b_{E,\mathit{\mathsf{g}}}}\right)}\right)}^2\times {\left(\frac{\widehat{b_{T,\mathit{\mathsf{g}}}}}{SE\left(\widehat{b_{T,\mathit{\mathsf{g}}}}\right)}\right)}^2\right)$ where the maximum was over all gene expression measurements and $\overline{v_{\mathit{\mathsf{g}}}^{\ast }}$ was the average of all ${\nu}_{\mathit{\mathsf{g}}}^{*}$.

## Results and discussion

The results for this GAW19 working group varied widely as a result of the different objectives of each contributor. Table 2 provides a brief summary of specific results.

Under *H*_0_, extreme *p* values follow a beta distribution [47]. Almeida et al [36] reported that the beta distribution provided an excellent fit to determine the effective number of independent tests *n*_*e*_ for *n* single-locus tests. For whole genome sequence, $\frac{n_e}{n}=15\%$; that is, accounting for LD reduced the multiple-testing burden by 85 %. However, significant associations could only be found when LD-correcting the significance level after a priori reducing sequence data based on functional annotations. Then 2 SNPs were detected: rs218966 in gene *PHF14* associated with SBP and rs9836027 in *MAP4* associated with DBP.

Liu et al [37] scanned chromosome 3 (GWAS data) for IBD sharing segments that associated with DBP. No genome-wide significance was found. However, several risk variants were detected in the region of gene *ZPLD1* by using CADD functional scores and sequence for the most promising region at 3q12.3.

**Fig. 2:**
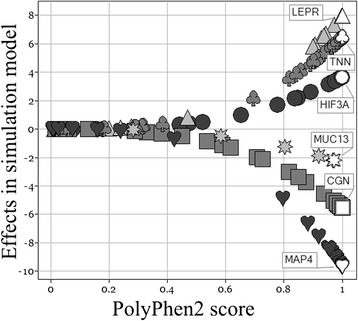
SNV effect sizes on GAW19 simulated DBP increase with increasing PolyPhen2 scores. Depicted are 6 genes with a range of SNV effect sizes that could be simultaneously displayed. Symbols depict SNVs in the same gene: LEPR (▲), TNN (♣), HIF3A (●), MAP4(♥), MUC13(✷), CGN(■)

**Fig. 3:**
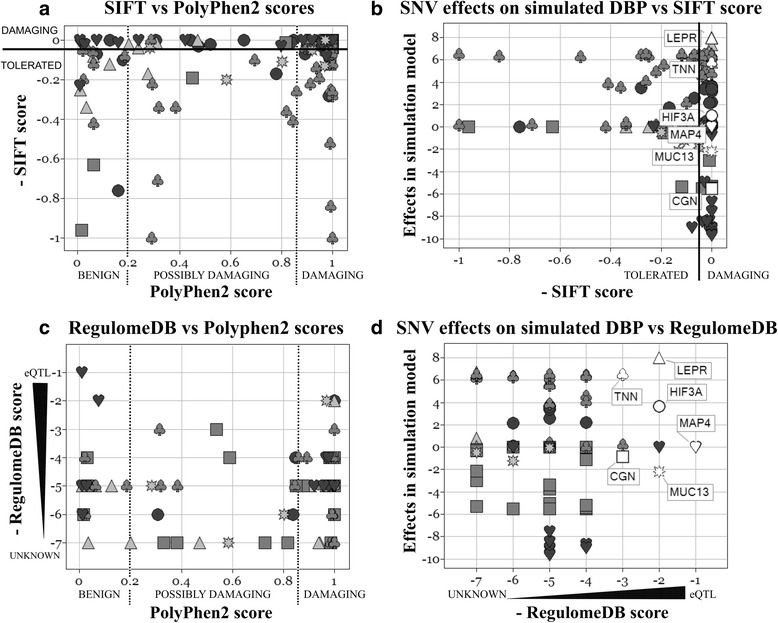
Comparison between the PolyPhen2, SIFT, and RegulomeDB functional prediction scores. *Left column*: Correlation of PolyPhen2 functional prediction scores with (**a**) SIFT or (**c**) RegulomeDB scores. Functional scores were transformed to have the same directionality. Nonsynonymous coding SNVs that alter the protein function should receive a PolyPhen2 score of 1 and a SIFT score of 0. Scores are metric and can be categorized as displayed. RegulomeDB annotates regulatory SNVs by an ordinal score ranging from the highest evidence (eQTL, expression quantitative trait locus) to the lowest. *Right column*: Filters or *priors* based on (**b**) SIFT or (**d**) RegulomeDB functional scores are partially mismatched on GAW19 simulated DBP. Symbols depict SNVs in the same gene: LEPR (**▲**), TNN (**♣**), HIF3A (**●**), MAP4(**♥**), MUC13(✷), CGN(**■**)

In the GAW19 trait simulation model, SNV effect sizes were based on PolyPhen2 functional prediction scores (Fig. 2) [35]. In Figs. 2 and 3, displayed SNV effects, PolyPhen2 scores, and the assignment to positions and genes (NCBI build37, human genome build 19) came from the simulation answers. To illustrate differences between functional annotations, SIFT scores (and rs-numbers) were added by annotating sequence (variant call format [vcf] files) with ANNOVAR and merging vcf files and simulation answers by chromosome and position. RegulomeDB scores were merged by dbsnp138 rs-identifier. Furthermore, functional scores were transformed to have the same directionality (Fig. 3). Different functional annotations focus on different information about SNVs and only annotate selected SNVs. PolyPhen2 and SIFT both annotate nonsynonymous coding SNVs by a metric score that can be categorized to distinguish benign mutations from damaging ones affecting protein function. Nevertheless, PolyPhen2 and SIFT scores differ to a substantial extent in value and category (Fig. 3a). RegulomeDB annotates regulatory SNVs by an ordinal score ranging from the highest evidence (eQTL, expression quantitative trait locus) to the lowest. Figure 3c illustrates that some SNVs were rated to affect gene expression and transcription factor binding (RegulomeDB scores 1 to 5) but not the protein function (scored “benign” by PolyPhen2). For *simulated* BP, SIFT and RegulomeDB annotations yield mismatched filters or *priors* whenever they deviate from the PolyPhen2 score used to simulate SNV effects. For example, SIFT annotated some SNVs with large effects in gene *TNN* as benign mutations (Fig. 3b) and only few SNVs in associated genes were rated to be of regulatory importance (Fig. 3d). Nevertheless, for *real* SBP, several multiple-testing adjusted significant windows (2 with SKAT, 4 with burden test T5) were only found when including RegulomeDB scores as variant weights for rare SNV analysis [27]. One of these regions contained *SNUPN* [27] which is a novel finding not previously reported to associate with BP. T5 and SKAT maintained the nominal significance level on simulated unassociated trait Q1 also when incorporating RegulomeDB scores into variant weights [27]. Kim and Wei [27] and Zhang et al [28] both recommended using relatively big differences in SNV weights distinguishing functional from nonfunctional SNVs. Zhang et al [28] observed that different burden tests with functionally informative SNV weights yielded different top ranked genes. Although no gene was significant, many of them had been reported in the BP literature before. For SKAT, Malzahn et al [30] found that variant weights, but not kernel choice, had a strong influence on power, for rare as well as common SNVs. Kernel methods may gain power by exploiting SNV correlations. This can be utilized fully by analyzing LD blocks [30]. LD structure also influenced which strategy yielded the best joint test of rare and common SNVs with SKAT [30].

When using gene expression data to informatively weight gene-wise *p* values for association of rare SNV burden with BP [33], 153 genes (out of 6118) reached nominal significance (weighted *p* ≤0.05). *P* value weights were determined such that evidence for phenotype associated gene expression lowered burden test *p* values. As no gene reached multiple-testing adjusted significance, Ho et al [33] used gene set enrichment analysis as aggregation test to relate the 153 top genes to biological pathways.

## Conclusions

All analyses presented herein used a cross-sectional design by analyzing trait data of the first examination, the first available examination, or longitudinally averaged traits. This mainly contributed to differences in sample size and trait variability. Furthermore, analyzing trait values at different time points may affect the marginal effect of genes that interact with age.

Including biological knowledge increased the power of association studies performed in our GAW group; especially filtering variants based on putative functional relevance. *Prior* weights can be included at different stages of the testing procedure. They can be incorporated into the test statistic of SKAT or burden tests, used when combining test statistics, or applied to association test p values. Selecting variant-sets also should take genetic structures into consideration, such as LD or IBD sharing. Moreover, the effective number of independent tests can be determined relatively easily by extreme value theory. This enables appropriate adjustment of the significance level for multiple testing to avoid an overly conservative approach. Ideally, variant grouping and selection, inclusion of biological information, and significance level adjustment can be applied simultaneously. Strategies like these are useful in increasing power in analyses of highly dense genetic data sets.

Filtering variants clearly boosted power in the discussed studies. However, filtering might also lose information. Functional scores such as PolyPhen2, SIFT, CADD, or RegulomeDB differ as they focus on different information about SNVs. Moreover, appropriateness of functional scores for a considered trait is a priori unknown. Hence, one is well advised to use and combine multiple functional annotations into a single filter or *prior*. This is feasible as functional annotations yield strong filters that greatly reduce the SNV space.
